# MF-300 (15-PGDH ENZYME INHIBITOR) REVERSES AGE-RELATED MUSCLE WEAKNESS IN MICE BY RESTORING MUSCLE QUALITY

**DOI:** 10.1093/geroni/igae098.3672

**Published:** 2024-12-31

**Authors:** Micah Webster, Jennifer Martin, Bruce Fahr, Ramzi Khairallah

**Affiliations:** Epirium Bio, San Diego, California, United States; Myologica LLC, Baltimore, Maryland, United States; Epirium Bio, San Diego, California, United States; Myologica LLC, Baltimore, Maryland, United States

## Abstract

Sarcopenia is an age-related disease that causes muscle weakness and reduced muscle quality with disproportionate impact on fast-twitch muscle. Gene expression of 15-hydroxyprostaglandin dehydrogenase (15-PGDH), an enzyme that metabolizes prostaglandins including PGE2, is elevated in older adult muscle and may contribute to sarcopenia. The study tested whether MF-300, a 15-PGDH inhibitor, could improve muscle quality (force per unit of mass, defined as specific force) in an ageing mouse model. C57BL/6J mice, aged 90-92 weeks, were stratified on body weight and muscle force into groups that were administered oral MF-300 (10, 30 or 60mg/kg every other day) or vehicle (control). A group of 39-54 week-old mice administered vehicle served as a younger adult comparator. Nerve evoked force production of the plantar flexors was measured and normalized to muscle mass to assess muscle specific force. Aged-vehicle mice had lower absolute and specific force relative to the adult-vehicle group. After 12 weeks of treatment, MF-300 10mg/kg significantly increased maximal muscle force (p< 0.0001) and specific force (p=0.02) in aged animals compared to vehicle. MF-300 10 mg/kg showed a significant increase in absolute (p=0.0003) and specific muscle force (p=0.02), as well as contraction rate (p=0.0068), of the extensor digitorum longus (a fast-twitch muscle) compared to the aged-vehicle group. This experiment demonstrates that MF-300 enhances the intrinsic properties of aged fast twitch muscle fibers independently of muscle mass. These findings suggest that MF-300 holds potential as therapeutic strategy for restoring muscle quality and strength in sarcopenia.

